# Structure and possible function of a G-quadruplex in the long terminal repeat of the proviral HIV-1 genome

**DOI:** 10.1093/nar/gkw432

**Published:** 2016-06-13

**Authors:** Beatrice De Nicola, Christopher J. Lech, Brahim Heddi, Sagar Regmi, Ilaria Frasson, Rosalba Perrone, Sara N. Richter, Anh Tuân Phan

**Affiliations:** 1 School of Physical and Mathematical Sciences, Nanyang Technological University, Singapore; 2 Department of Molecular Medicine, University of Padua, Italy

## Abstract

The long terminal repeat (LTR) of the proviral human immunodeficiency virus (HIV)-1 genome is integral to virus transcription and host cell infection. The guanine-rich U3 region within the LTR promoter, previously shown to form G-quadruplex structures, represents an attractive target to inhibit HIV transcription and replication. In this work, we report the structure of a biologically relevant G-quadruplex within the LTR promoter region of HIV-1. The guanine-rich sequence designated *LTR-IV* forms a well-defined structure in physiological cationic solution. The nuclear magnetic resonance (NMR) structure of this sequence reveals a parallel-stranded G-quadruplex containing a single-nucleotide thymine bulge, which participates in a conserved stacking interaction with a neighboring single-nucleotide adenine loop. Transcription analysis in a HIV-1 replication competent cell indicates that the *LTR-IV* region may act as a modulator of G-quadruplex formation in the LTR promoter. Consequently, the *LTR-IV* G-quadruplex structure presented within this work could represent a valuable target for the design of HIV therapeutics.

## INTRODUCTION

G-quadruplexes are nucleic acid secondary structures that may form in G-rich sequences under physiological conditions (1–3). In contrast to duplex DNA formed by Watson-Crick base-pairing, the building blocks of G-quadruplexes are stacked guanine tetrads (G-tetrads) assembled by Hoogsteen-type base-pairing. The presence of coordinating cations is important to G-quadruplex formation and stability (4–6). G-quadruplex structures are highly polymorphic, both in terms of strand stoichiometry (forming inter- and intramolecular structures) and strand orientation/topology (7,8). G-quadruplex-forming motifs have been found in telomeres, G-rich micro- and mini-satellites and near oncogene promoters (8–17). Human G-quadruplex DNA motifs have been reported to be associated with recombination prone regions (18) and to show mutational patterns that preserved the potential to form G-quadruplex DNA structures (14). Expansion of G-quadruplex-forming motifs has been associated with relevant human neurological disorders (19–25). The identification of G-quadruplex binding proteins (26,27) and their visualization in cells with antibody-based technology (28,29) have also provided convincing evidence of the existence of G-quadruplexes *in vivo*.

Recently, the presence of G-quadruplexes in viruses and their involvement in viral key steps have also been reported (30). G-quadruplexes have been implicated in pathogenic mechanisms of the Epstein-Barr virus (31,32), SARS coronavirus (33), herpes simplex virus 1 (34) and the human papillomavirus (35). We and other groups have identified functionally significant G-quadruplexes in the Nef coding region (36) and the unique long terminal repeat (LTR) promoter (37–39) of the human immunodeficiency virus (HIV), the etiologic agent of the acquired immune deficiency syndrome (AIDS), a major worldwide epidemic.

HIV establishes a persistent infection in human hosts, with the depletion of CD4+ lymphocytes, the major target cells of viral infection *in vivo*, eventually resulting in defective cellular immunity, and thus leading to full-blown AIDS. The Joint United Nations Programme on HIV/AIDS (UNAIDS) estimates that there were 35.3 million people living with HIV at the end of 2012 compared with 26.2 million in 1999 – a 35% increase. Although important progress has been achieved in preventing new HIV infections and prolonging HIV patients’ life with antiretroviral therapy, the infection cannot be eradicated and therefore AIDS-related illnesses are projected to continue as a significant worldwide cause of premature mortality in the coming decades if a decisive cure is not found (40).

**Figure 1.**
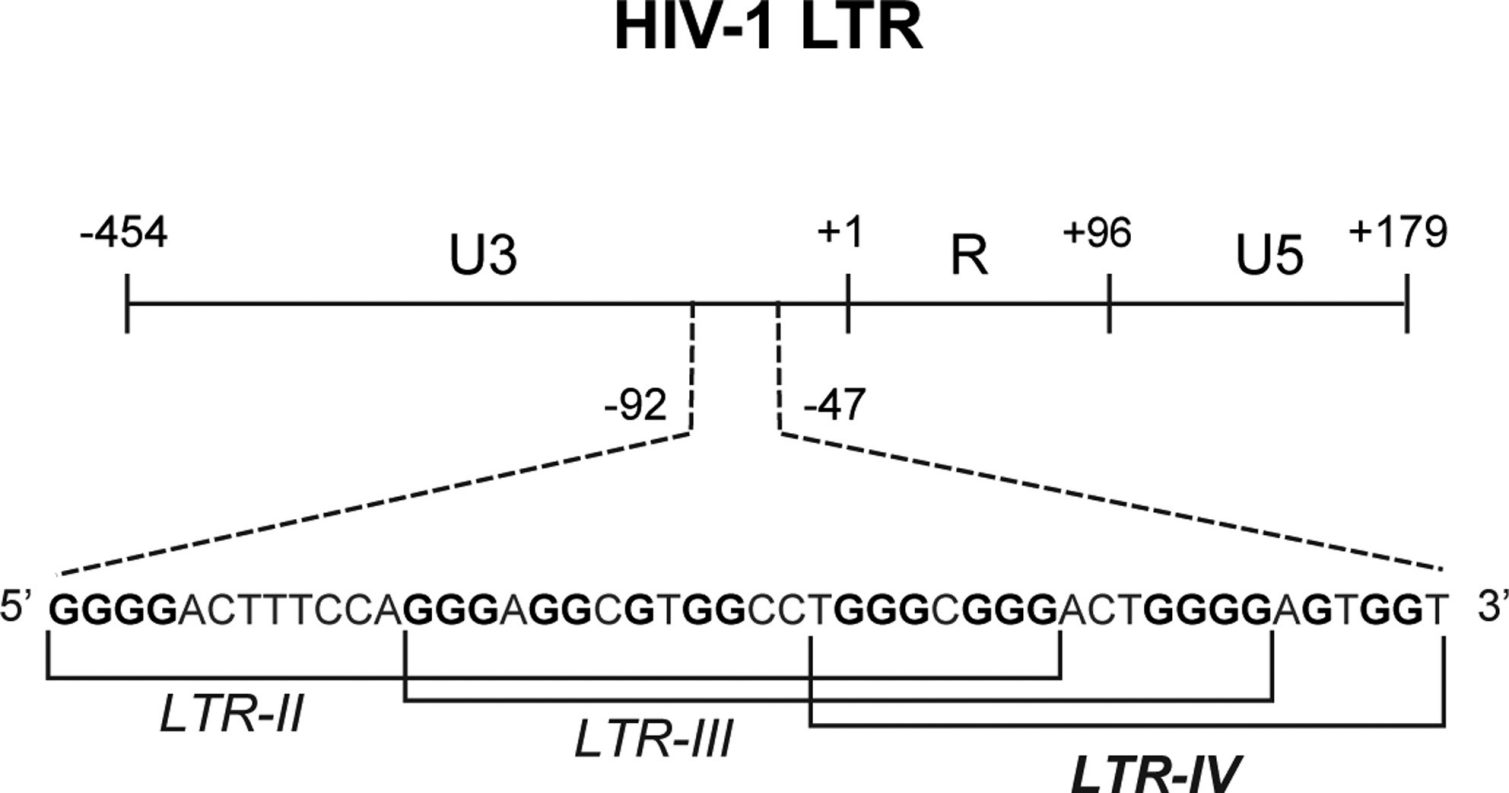
Sequence of the G-rich region embedded in the U3 moiety of the LTR promoter in the proviral HIV-1 genome. The *LTR-IV* sequence is the focus of this work. The *LTR-III* and *LTR-II* regions are also shown.

HIV is composed of two copies of positive single-stranded RNA that codes for the nine virus genes. The genomic RNA is reverse transcribed into a linear double-stranded DNA molecule that is next integrated into the human chromosome by the viral enzyme integrase. The stably integrated HIV-1 provirus, a 10 kb-long DNA molecule, serves as a template for the transcription of viral messengers and genomic RNA by the cellular Pol II polymerase (41). Viral transcription is triggered by the interaction of cellular transcription factors with the U3 region of the LTR promoter (Figure 1) (42). The LTR promoter contains multiple G-rich sequences that have been shown to adopt G-quadruplex structures. The formation of G-quadruplexes in the LTR results in decreased viral transcription (37), with an effect that is enhanced by the presence of the cellular protein nucleolin (43) and G-quadruplex ligands (44).

Interestingly, within the G-rich region of the LTR promoter one of the LTR G-quadruplexes, termed *LTR-IV*, does not readily form in physiological ionic conditions. However, *LTR-IV* can be induced by a G-quadruplex stabilizing ligand (37), making it a good G-quadruplex targets for antiviral therapy.

Several G-quadruplex ligands have been developed against cellular G-quadruplexes implicated in tumor pathogenesis; at least two molecules have also been tested and proved effective as antiviral agents against HIV (36,44). Recently, a few compounds selective for HIV-1 LTR G-quadruplexes have also been reported (45). These studies have shown the potential of developing antiviral molecules with a G-quadruplex-mediated mechanism of action. To increase the selectivity of G-quadruplex binding ligands toward a viral target over other G-quadruplexes that may form within the cell, the high-resolution structure of the target G-quadruplexes will allow the rational design and virtual screening of molecules with selective binding. Considering the value of such target elucidation, we report on the nuclear magnetic resonance (NMR) solution structure of a G-quadruplex formed by the 22-nt G-rich *LTR-IV* sequence (5′-CTG_3_CG_3_ACTG_4_AGTG_2_T-3′) from the promoter region of the HIV-1 LTR.

## MATERIALS AND METHODS

### DNA sample preparation

Oligonucleotides used in the *Taq* polymerase stop assay and in the construction/mutagenesis of the plasmids for the luciferase reporter assay were purchased from Sigma Aldrich (Milan, Italy). All other DNA oligonucleotides were purchased from Integrated DNA Technologies (Coralville, IA, USA) or chemically synthesized on an ABI 394 DNA/RNA synthesizer. Synthesized oligonucleotides were purified and dialyzed successively against 20 mM KCl solution and water before being lyophilized. DNA concentration was expressed in strand molarity using a nearest-neighbor approximation for the absorption coefficients of the unfolded species (46).

### Gel electrophoresis

The molecular size of G-quadruplexes was visualized by non-denaturing polyacrylamide gel electrophoresis (PAGE). Samples were incubated in 10 mM potassium phosphate buffer (pH 7) before loaded on 20% polyacrylamide gels with 40% (v/v) sucrose added before loading. Gels were run with 10 mM potassium phosphate buffer (pH 7) at room temperature for 90 min at 120 volts. DNA migration was imaged using UV-shadowing. For *Taq* polymerase stop assay, the DNA extension products were separated on a 15% denaturing gel (7 M urea), run in TBE buffer for 3 h at 80 W, and finally visualized by phosphorimaging (Typhoon FLA 9000).

### Circular dichroism

Circular dichroism (CD) spectra were recorded on a JASCO-815 spectropolarimeter using 1-cm path length quartz cuvettes. DNA samples (strand concentration, 5 μM) were dissolved in a buffer containing 70 mM potassium chloride and 20 mM potassium phosphate (pH 7) in a reaction volume of 500 μL. For each experiment, an average of three scans was taken, the spectrum of the buffer was subtracted, and the data were zero-corrected at 320 nm.

### Thermal denaturing

The thermal denaturing of the *LTR-IV* G-quadruplex was monitored by UV absorption on a JASCO V-650 UV-Vis spectrophotometer. DNA concentration was 5 μM. Samples contained 70 mM potassium chloride and 20 mM potassium phosphate, pH 7. Absorbance was monitored at 295 nm and zero-corrected at 320 nm, over the temperature range from 20 to 90°C. Melting curves were baseline-corrected to compute the fraction of folded G-quadruplex over varying temperature. The melting temperature was determined as the temperature at which half the population was in the folded state.

### NMR spectroscopy

NMR experiments were performed on 600 and 700 MHz Bruker spectrometers at 25°C. DNA samples (strand concentration, 0.2−1.5 mM) were dissolved in a buffer containing 70 mM potassium chloride and 20 mM potassium phosphate (pH 7). Resonances for guanine residues were assigned unambiguously by using site-specific low-enrichment ^15^N labeling (47), site-specific ^2^H labeling (48) and through-bond correlations at natural abundance (49). Spectral assignments were completed by NOESY, TOCSY, (^13^C-^1^H)-HMBC and (^13^C-^1^H)-HSQC as previously described (50). Inter-proton distances were deduced from NOESY experiments at various mixing times. All spectral analyses were performed using the SPARKY program (51).

### Structure calculation

Structures of the *LTR-IV* G-quadruplex were first calculated by performing distance geometry simulated annealing using the XPLOR-NIH program (52). The ten lowest-energy structures were then used for further refinement via molecular dynamics (MD) simulations using the AMBER 10 program (53). Prior to MD simulation, K^+^ ions were introduced around the structure computed in XPLOR, including internal placement between G-tetrads of the G-quadruplex. Structures were then solvated before undergoing a series of constrained minimization and simulation steps as previously described (54). Finally, structures were refined over 1 ns of restrained MD simulations including hydrogen-bond restraints and inter-proton distance restraints. Inter-proton distances were deduced from NOESY experiments performed in H_2_O (mixing time, 300 ms) and ^2^H_2_O (mixing times, 100 and 300 ms). The glycosidic dihedral restraints were based on intra-residue NOEs of H1′-H6/8 cross-peak intensities observed at the mixing time of 100 ms.

### Data deposition

The coordinates of the *LTR-IV* G-quadruplex have been deposited in the Protein Data Bank under accession code 2N4Y.

### Plasmid construction

The wild-type (*WT*) HIV-1 LTR region (corresponding to segment −381/+83 in the HIV-1 genome) was inserted into the promoterless luciferase reporter vector pGL4.10-Luc2 (Promega Italia, Italy), as previously reported (37). The *G18T* mutant pGL4.10-Luc2/LTR vector was generated using QuikChange mutagenesis kit (Stratagene/Agilent Technologies) with the following primers: PrMut *G18T* (i) 5′-CCTGGGCGGGACTGGGGA**T**TGGCGAGCCCTCAGATCC-3′ and PrMut *G18T* (ii) 5′-GGATCTGAGGGCTCGCCA**A**TCCCCAGTCCCGCCCAGG-3′, where the mutated base is shown in boldface and underlined. Both the *WT* and *G18T* LTR sequences were confirmed by sequencing. Plasmid size was confirmed by Hind III linearization on 1% agarose gel.

### Reporter assay

Luciferase activity of the *WT* and *G18T* mutant LTRs was assessed in human embryonic kidney 293T (HEK293T) cells seeded in 12-well plates (2 × 10^5^ cells/well). Cells were transfected 24 h post-seeding with pGL4.10-LTR-*WT* or pGL4.10-LTR-*G18T* (100 ng/well) using TransIT-293 transfection reagent (Mirus Bio LLC, Madison, WI, USA), according to the manufacturer's protocol. After 30 min, cells were treated with increasing concentrations (1–4 μM) of BRACO-19 (ENDOTHERM, Saarbruecken, Germany) for 24 h. Luciferase activity was measured using the britelite plus Reporter Gene Assay System (PerkinElmer Inc., Milan, Italy) in a Victor X2 multilabel plate reader (PerkinElmer Inc., Milan, Italy). Cells were lysed in lysis buffer (1X PBS, 1% TRITON X-100) and protein concentration was determined by bicinchoninic acid (BCA) assay (Thermo Scientific Pierce, Monza, Italy). Luciferase signals were subsequently normalized to total protein content, according to the manufacturer's protocol (http://ita.promega.com/∼/pdf/resources/pubhub/cellnotes/normalizing-genetic-reporter-assays/). All experiments were performed twice and in duplicate.

HEK 293T cells sustain all viral steps with the exception of virion attachment and entry as they lack cell receptors recognized by HIV-1. They can be transfected with the HIV-1 proviral genome to produce fully infectious viral particles, indicating that their cytoplasmic/nuclear protein makeup is capable of sustaining viral transcription and replication (55).

### *Taq* polymerase stop assay

*Taq* polymerase stop assay was carried out as previously described (37). Briefly, the 5′-end labeled primer (5′-GGCAAAAAGCAGCTGCTTATATGCAG-3′) was annealed to the templates (LTR-II+III+IV *WT* Taq 5′-TTTTTGGGGACTTTCCAGGGAGGCGTGGCCTGGGCGGGACTGGGGAGTGGTTTTTCTGCATATAAGCAGCTGCTTTTTGCC-3′ or LTR-II+III+IV *G18T* Taq 5′-TTTTTGGGGACTTTCCAGGGAGGCGTGGCCTGGGCGGGACTGGGGA**T**TGGTTTTTCTGCATATAAGCAGCTGCTTTTTGCC-3′) in lithium cacodylate buffer in the presence/absence of 100 mM KCl by heating to 95°C for 5 min and gradually cooling to room temperature. Where specified, samples were incubated with BRACO-19 (62.5–250 nM). Primer extension was conducted with 2 U of AmpliTaq Gold DNA polymerase (Applied Biosystem, Carlsbad, California, USA) at 37°C for 30 min. The marker sample was obtained by treating the elongation *WT* product with the sequencing protocol of Maxam *et al*. (56). Reactions were stopped by ethanol precipitation, primer extension products were separated on a denaturing gel and visualized by phosphorimaging (Typhoon FLA 9000). The intensities of stop bands were quantified using ImageQuant TL software (GE Healthcare Europe, Milan, Italy).

### Analysis of G18 conservation

Conservation of the G18 base involved in the *LTR-IV* G-quadruplex formation was evaluated by aligning 953 LTR U3 sequences of different HIV-1 subtypes including 24 subtypes A, 485 subtypes B and 119 subtypes C from the HIV Sequence Database (http://www.hiv.lanl.gov/) using Jalview (http://www.jalview.org/). The analyzed sequences belong to a broad spectrum of HIV-1 subtypes, both non-recombinant subtypes of Group M and circulating recombinant forms.

## RESULTS

### Formation of a monomeric parallel G-quadruplex by the *LTR-IV* sequence

**Figure 2.**
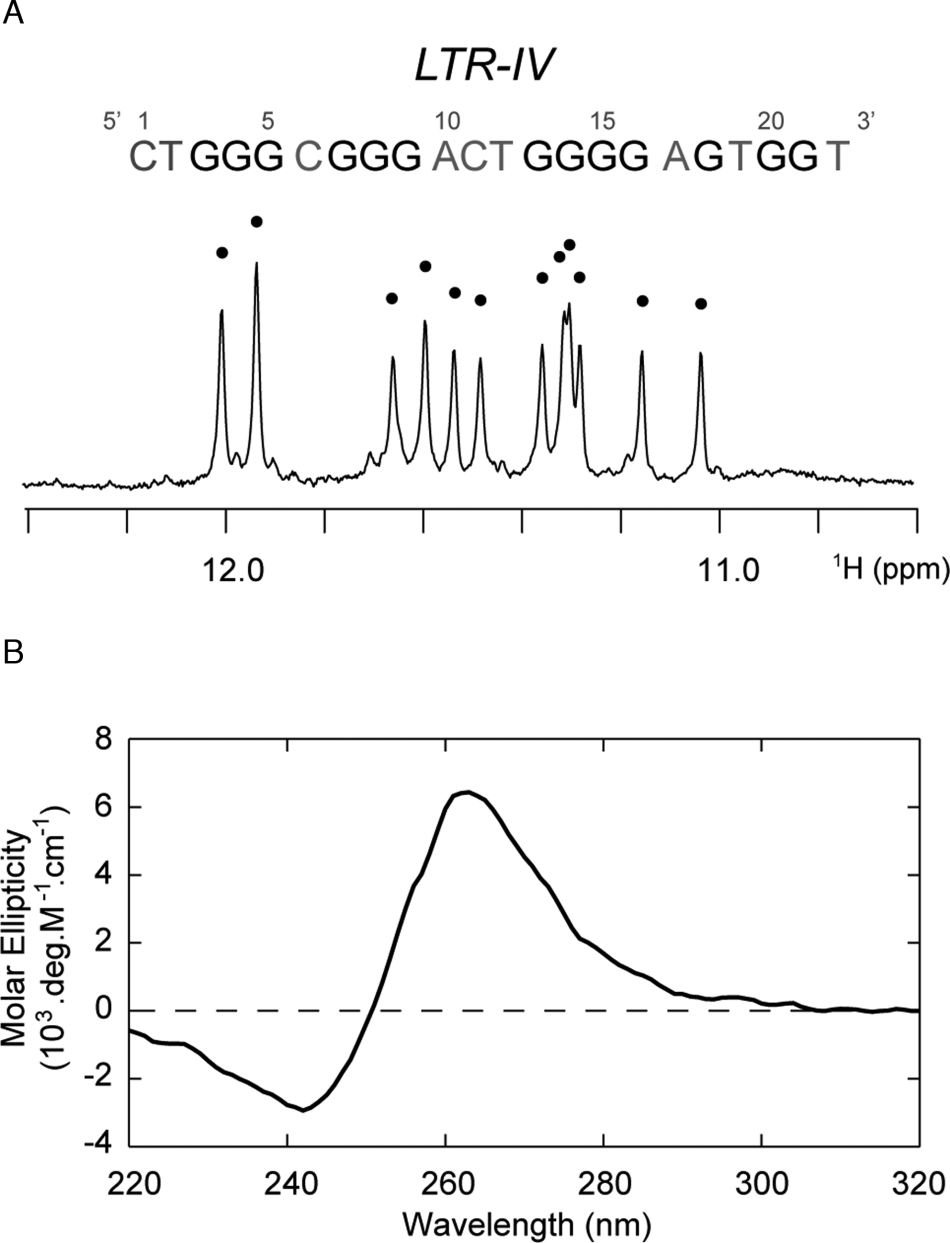
Biophysical characterization of the *LTR-IV* sequence. (**A**) NMR imino proton spectrum and (**B**) CD spectrum of *LTR-IV* suggest the formation of a major parallel-stranded G-quadruplex conformation.

The NMR spectrum of the *LTR-IV* sequence displays 12 well-resolved peaks in the imino proton region (Figure 2A), indicating the formation of a three-layer G-quadruplex structure. The CD spectrum of this sequence, showing a negative peak at 240 nm and a positive peak at 260 nm (Figure 2B), is indicative of a parallel G-quadruplex folding topology. Non-denaturing PAGE experiments show two separate species (Supplementary Figure S1): a major form that migrates at a rate consistent with a monomeric G-quadruplex reference and a small population of higher-ordered structure. Solvent exchange with ^2^H_2_O reveals slow exchange rates for four imino protons (Supplementary Figure S2) – corresponding to a single central G-tetrad layer. A single transition was observed in the thermal denaturing curve of *LTR-IV* (Supplementary Figure S3) showing a melting temperature of 50.5°C in ∼100 mM K^+^. Collectively, these biophysical data suggest that *LTR-IV* predominantly forms a major G-quadruplex species that adopts a monomeric parallel-stranded conformation.

### *LTR-IV* adopts a parallel G-quadruplex containing a T-bulge

**Figure 3.**
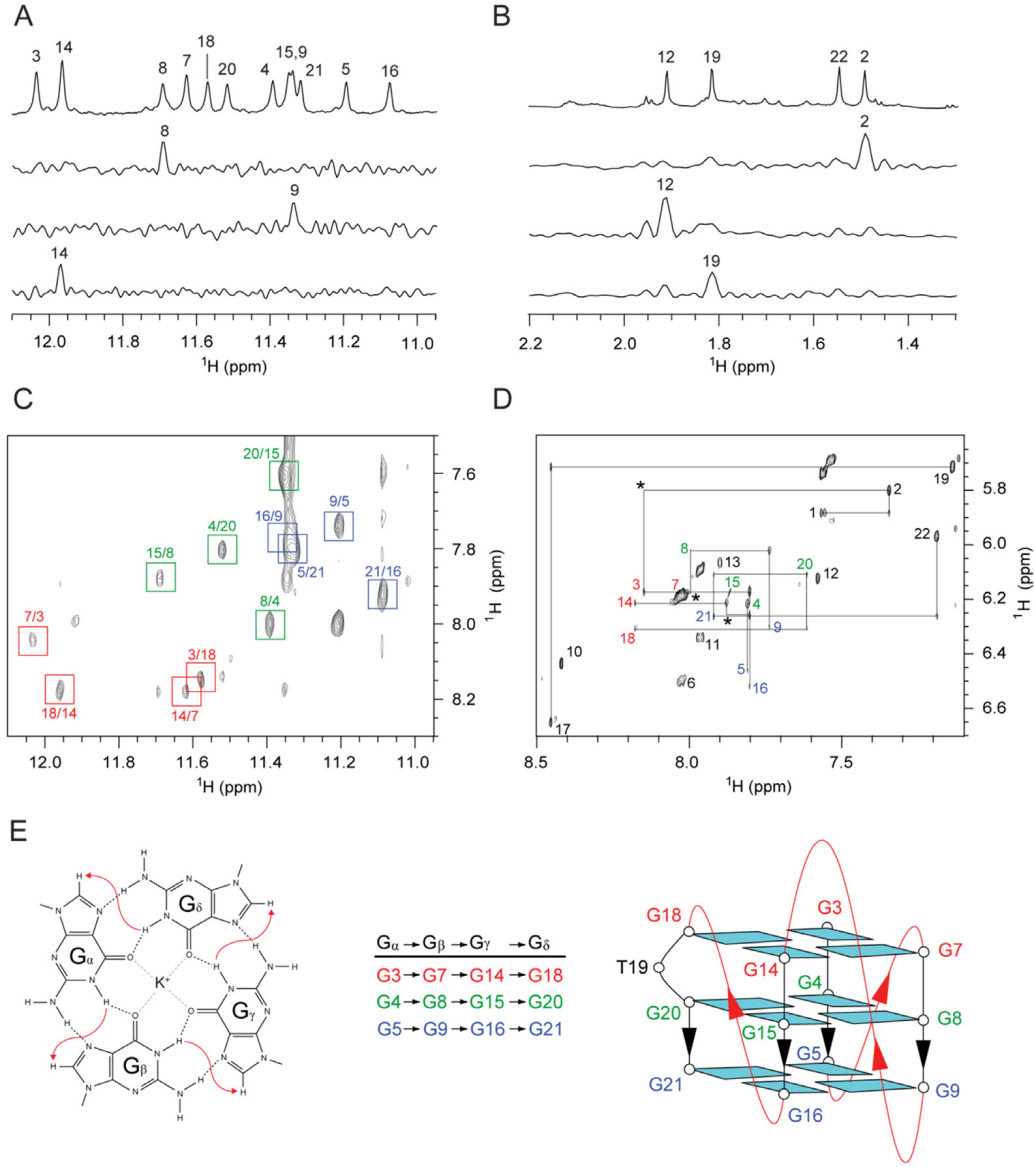
NMR determination of the *LTR-IV* folding topology. (**A**) Reference imino proton spectrum of *LTR-IV* with examples of ^15^N-filtered spectra of site-specific 4% ^15^N-enriched samples. (**B**) Reference methyl proton spectrum with examples of ^13^C-filtered spectra of sequences containing site-specific ^13^C-enriched thymine substitutions. (**C**) Guanine H1-H8 cyclical connectivities in NOESY spectra. Cross-peaks are colored based on G-tetrad arrangement. (**D**) Tracing the H8/6-H1' NOE sequential connectivity in NOESY spectrum (mixing time, 300 ms). Intraresidue H8/6-H1′ cross-peaks are labeled with residue numbers and color coded based on G-tetrad arrangement. Missing cross-peaks are labeled with asterisks. (**E**) G-tetrad schematic highlighting the close proximity of H8-H1 protons used to determine guanine participation in G-tetrad formation. Folding topology of the *LTR-IV* sequence shows a parallel three-layered G-quadruplex conformation containing a T19 bulge.

We set out to elucidate the G-quadruplex structure of the *LTR-IV* sequence. Guanine imino (H1) protons were unambiguously assigned for all guanines by carrying out ^15^N-filtered experiments of sequences containing site-specific 4% ^15^N enrichment (Figure 3A and Supplementary Figure S4). Unambiguous assignment of guanine H8 protons was made by site-specific ^2^H labeling experiments (Supplementary Figure S4). Assignment of selected aromatic and methyl protons of thymine residues was made by through-bond correlation experiments, using site-specific 4% ^13^C,^15^N labeled samples (Figure 3B and Supplementary Figure S5). Based on these assignments, the H8/H6-H1′ sequence connectivity could be traced allowing for assignment of cross-peaks within NOESY spectra (Figure 3D).

Examining the cyclical through-space guanine H1-H8 cross peaks in NOESY spectra allowed the identification of guanine bases that make up the same G-tetrad (Figure 3C). H1-H8 cross peaks demonstrate the formation of three guanine tetrads, G3·G7·G14·G18, G4·G8·G15·G20 and G5·G9·G16·G21, indicating a parallel-stranded G-quadruplex fold is adopted by *LTR-IV* (Figure 3E). The location of the G4·G8·G15·G20 tetrad within the center of the structure is supported by the enhanced protection of the imino protons of guanines in this tetrad during solvent exchange experiments (Supplementary Figure S2). Guanine bases are shown to adopt an *anti* conformation, consistent with the intensity of their intra-residue H8-H1′ cross-peaks observed in NOESY spectra.

The resulting folding topology indicates three continuous tracts of guanines, participating in G-tetrad formation, G3–G5, G7–G9 and G14–G16, in addition to a fragmented tract consisting of G18, G20 and G21. This fragmented tract results in a bulge at the T19 position. These G-tracts are connected by loops including a short 1-nt propeller loop formed by C6, a 4-nt propeller loop formed by the A10-C11-T12-G13 sequence and another short 1-nt propeller loop formed by A17. The flanking sequence C1-T2 is situated on the 5′-end of the molecule, while the T22 base is found on the 3′-end.

### Solution structure of the *LTR-IV* G-quadruplex

**Figure 4.**
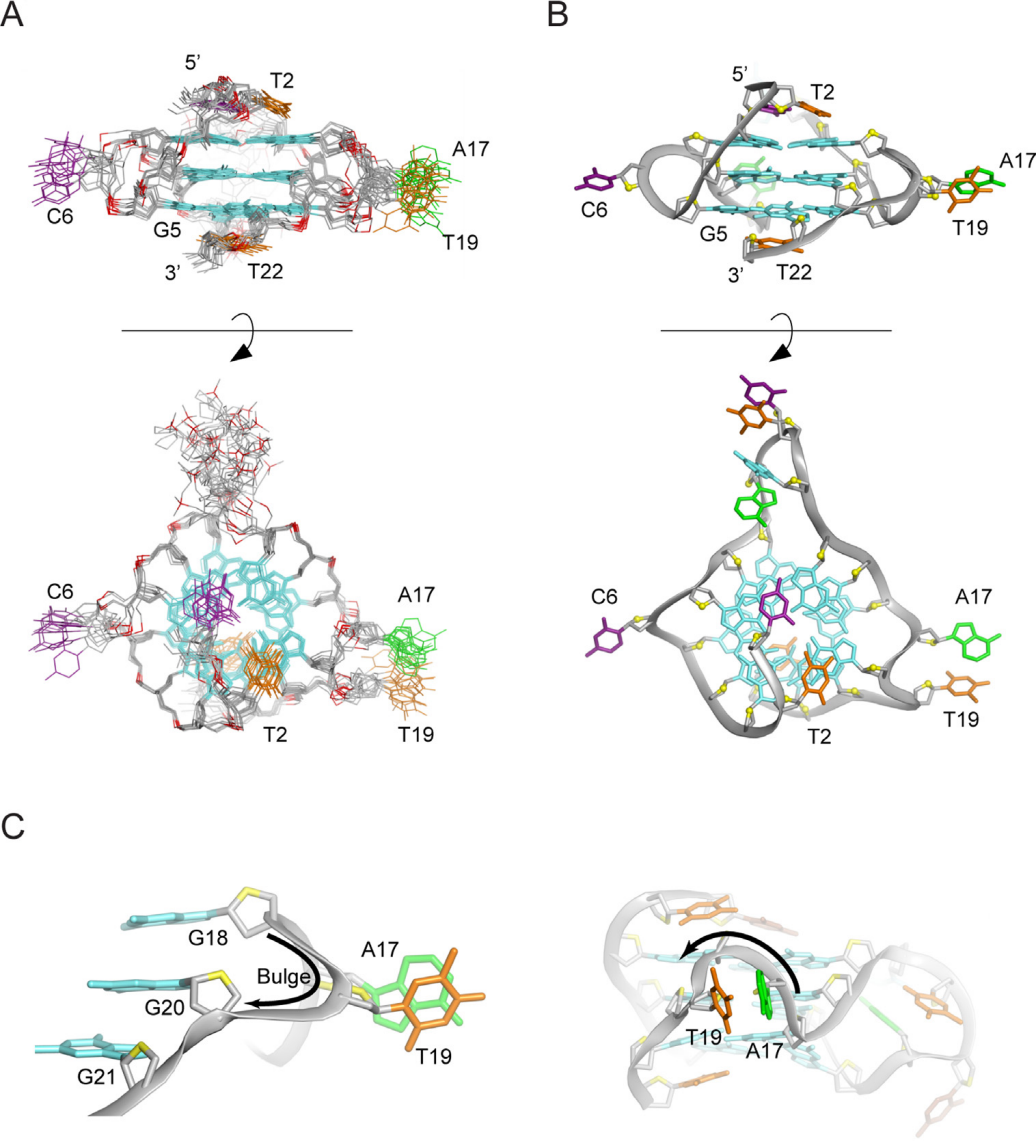
NMR solution structure of the *LTR-IV* G-quadruplex. (**A**) Alignment of the ten lowest-energy structures computed using XPLOR based on restraints from NMR experiments. Nucleobases of the 4-nt loop from positions 10–13 are omitted for clarity. (**B**) Ribbon view of a representative structure. (**C**) Close-up view of the T19 bulge and neighboring A17 propeller loop. Backbone are colored in grey. O4′ atoms of the sugar moiety are represented as yellow. Bases are colored as follows: guanine (cyan), adenine (green), thymine (orange) and cytosine (purple).

Structural calculation of the *LTR-IV* G-quadruplex was carried out based on restraints derived from NMR experiments (Table 1). The ten lowest-energy structures computed were well converged (Figure 4A and Table 1).

**Table 1.** Statistics of the computed structures of the *LTR-IV* G-quadruplex

**NMR restraints**		
Distance restraints	^2^H_2_O	H_2_O
intra-residue	402	4
sequential (i, i + 1)	93	12
long-range (i, ≥i + 2)	18	52
Other restraints		
hydrogen bond	48	
dihedral angle	12	
**Structure statistics**		
NOE violations		
number (>0.2 Å)	0.100 ± 0.300	
maximum violation (Å)	0.163 ± 0.061	
R.M.S.D. of violations (Å)	0.013 ± 0.002	
Deviations from ideal covalent geometry		
bond lengths (Å)	0.003 ± 0.000	
bond angles (°)	0.714 ± 0.025	
improper dihedrals (°)	0.370 ± 0.014	
Pair-wise all heavy atom R.M.S.D. values (Å)		
all heavy atoms of G-tetrad core	0.949 ± 0.174	
all heavy atoms excluding A10, T11, C12 and G13	1.398 ± 0.192	

The solution structure of *LTR-IV* shows interesting structural motifs (Figure 4B). (i) The T19 bulge readily stacks with A17 of the nearby 1-nt propeller loop. This stacking interaction is supported by multiple NOEs between the sugar and aromatic protons of these residues (Figure 3D). (ii) The 4-nt propeller loop is notably unstructured or highly dynamic, although this could potentially be due to lack of available constraints between the residues in this loop. (iii) The flanking C1 and T2 bases at the 5′-end of the molecule are located on top of the G3·G7·G14·G18 tetrad, partially capping the exposed tetrad. (iv) Similarly, T22 at the 3′-end caps the bottom G5·G9·G16·G21 tetrad. Collectively, these features give rise to a unique G-quadruplex structure containing a previously unreported loop-bulge stacking motif.

### Effect of mutations on the *LTR-IV* structure

We investigated how mutations affect the structure of the *LTR-IV* sequence. Specifically, we examined the role of the T19 bulge and the ability of the G-quadruplex to form using other combinations of guanine bases. We synthesized a series of sequences containing base substitutions and/or base deletions (Table 2) and examined resulting G-quadruplex formation in physiological K^+^ conditions. The sequence *G13T* containing a G-to-T substitution at position 13 confirms that G13 does not play an important role in the G-tetrad formation. The deletion of T19 in the *ΔT19* sequence tests the formation of G-quadruplex in the absence of a T19 bulge, creating a continuous GGG tract in its place. The sequence *G13TΔT19* addresses these two changes concurrently. We also checked the ability of the *LTR-IV* sequence to utilize other guanine bases in the formation of a G-quadruplex by substituting G16 and G18 with a thymine in the *G16T* and *G18T* sequences respectively.

**Table 2.** *LTR-IV* and mutated sequences

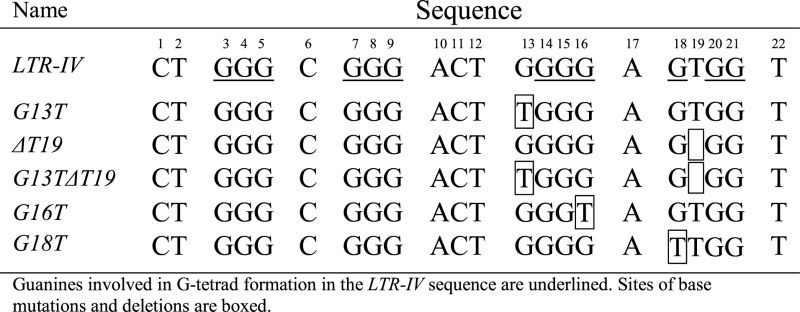									

**Figure 5.**
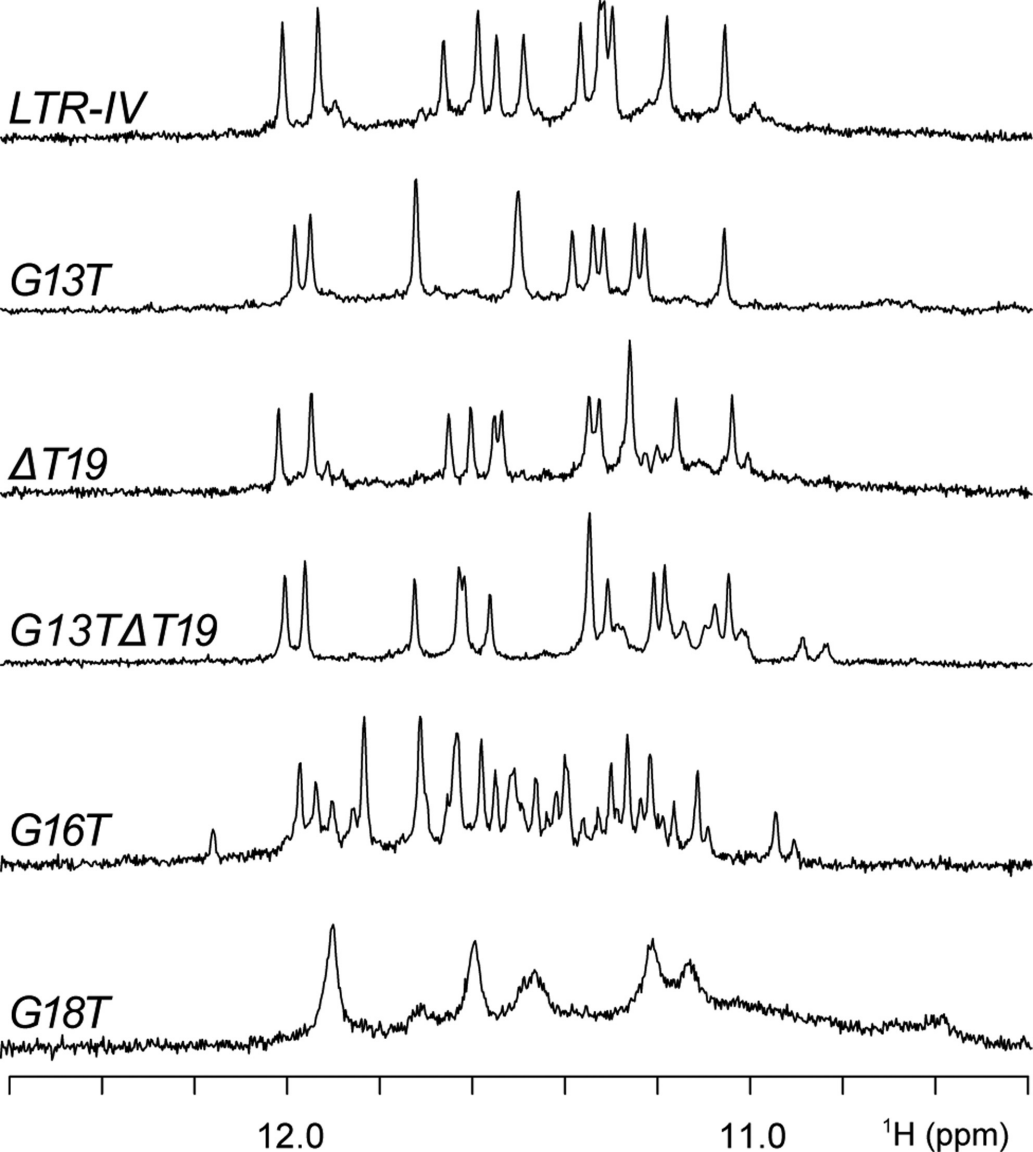
Imino proton NMR spectra of *LTR-IV* and sequences containing mutations and/or deletions in 70 mM potassium chloride and 20 mM potassium phosphate (pH 7).

Mutated sequences were analyzed using NMR to ascertain their ability to form G-quadruplex and to gauge the folding similarity based on their imino proton chemical shift pattern (Figure 5). The modification of G13 to T leads to small changes in the imino proton spectra of *G13T* but maintains the general chemical shift pattern, suggesting this guanine base is not critical to G-quadruplex formation. Similarly, the deletion of T19 in *ΔT19* results in a predominant G-quadruplex conformation with a similar imino proton chemical shift pattern to that of the unmodified *LTR-IV* sequence, although the emergence of a small minor population is visible. This suggests the T19 bulge is also not a key factor in the formation of a parallel G-quadruplex. The introduction of both of these modifications is still tolerated in *G13TΔT19*, although a slightly elevated level of a minor population is observed. Alternatively, substitution of G16 or G18 with thymine disrupted the folding topology: multiple conformations are adopted in the *G16T* sequence, while a different conformer is observed in the *G18T* sequence, displaying broad imino proton peaks. In addition to changes in NMR spectra, the mutated sequences *G16T* and *G18T* demonstrate markedly different CD spectra as compared to that of the unmodified *LTR-IV* sequence (Supplementary Figure S6) suggesting these mutations are highly disruptive to the native conformer. Collectively, these data demonstrated the T19-bulge is not mandatory for the formation of a parallel G-quadruplex from the *LTR-IV* region of the HIV-1 LTR. In contrast, the guanines in the G14-G16 and G18-G21 stretches are required.

### *LTR-IV* G-quadruplex formation correlates with HIV-1 LTR promoter activity

**Figure 6.**
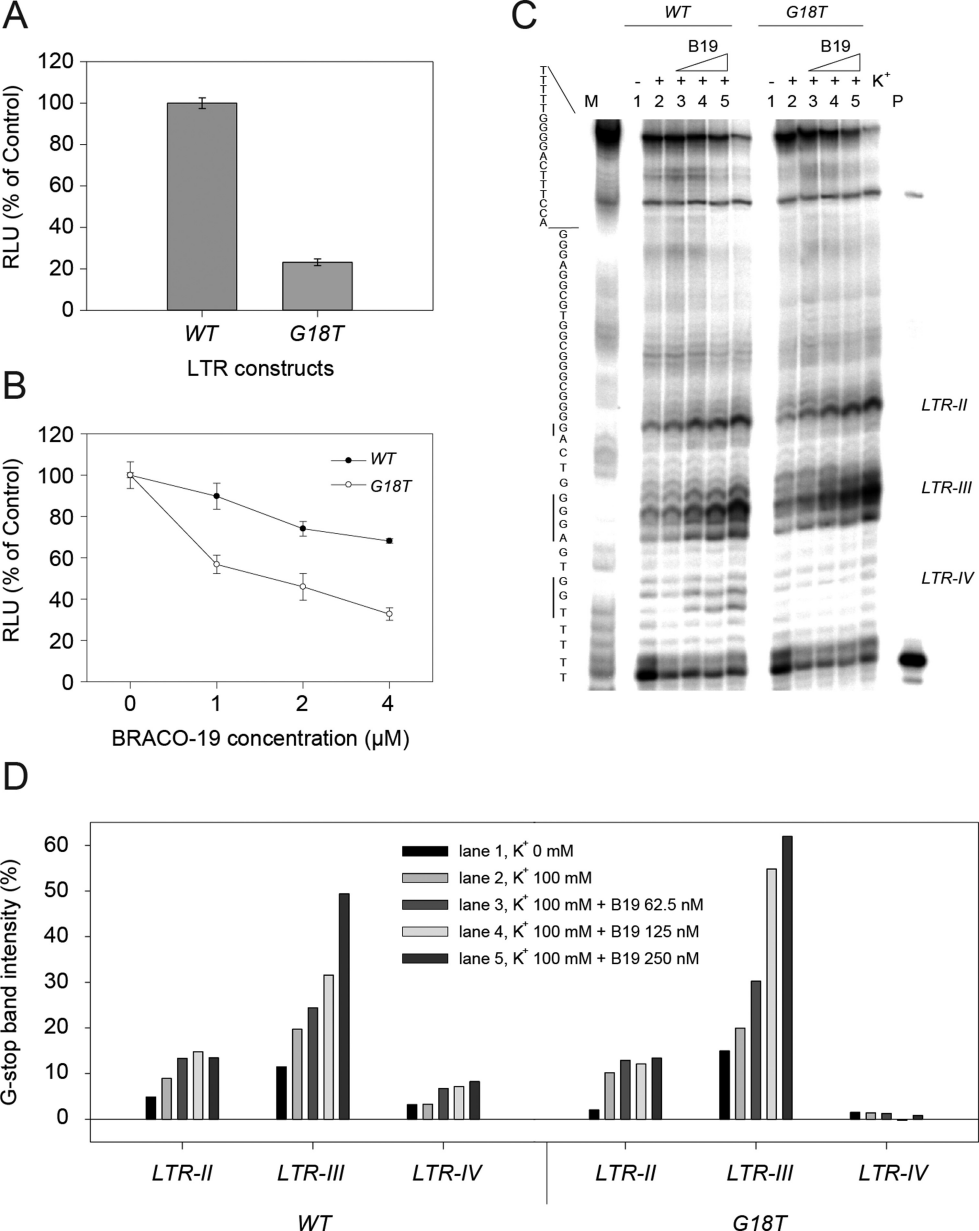
Biological effect of *LTR-IV* G-quadruplex. (**A**) Luciferase expression of the wild-type (*WT*) and mutant (*G18T*) *LTR-IV* normalized to total protein content in HEK 293T cells. (**B**) Normalized luciferase expression of the *WT* and mutant *G18T LTR-IV* in the presence of BRACO-19 (1.0–4.0 μM). (**C**) *Taq* polymerase stop assay performed in the absence or presence of K^+^ (100 mM) and BRACO-19 (B19) (62.5–250 nM) as indicated on the LTR-II+III+IV *WT* and mutant *G18T* templates. The *WT* sequence is shown on the left. Stop regions corresponding to *LTR-II, LTR-III* and *LTR-IV* are shown on the right and are indicated on the *WT* sequence. M is a marker lane and P indicates the lane where only the primer was loaded as control. (**D**) Quantification of the intensity of the stop bands obtained in the *Taq* polymerase stop assay. Stop band intensity was quantified by ImageQuant TL software (GE Healthcare Europe, Milan, Italy). This assay was run in duplicate, with consistent behavior observed across experiments.

To assess the physiological role of the *LTR-IV* G-quadruplex, a G18-to-T (*G18T*) mutation was introduced in the full-length HIV-1 LTR promoter fused to a luciferase reporter gene. As the G18T mutation was shown to abolish the *LTR-IV* G-quadruplex (Figure 5), we selected this sequence as a negative structural control. Both the wild-type (*WT*) and mutant *G18T* LTR-Luc plasmids were transfected into HEK 293T cells and the luciferase activity was measured. The promoter activity of the mutant *G18T* LTR was significantly reduced to 23% of the activity of the *WT* LTR (Figure 6A). Transfected cells were next treated with BRACO-19, a G-quadruplex ligand shown to bind and stabilize *LTR-III* and *LTR-IV* G-quadruplex structures (37). In the presence of the ligand, *WT* LTR promoter activity decreased to 68% of the untreated control at the highest tested ligand concentration (37), whereas the activity of the mutant *G18T* LTR decreased to an even further extent to 33% of the untreated control (Figure 6B).

To assess which G-quadruplex species form in the full-length LTR promoter, a *Taq* polymerase stop assay was set up. The *WT* and mutant *G18T* templates were subjected to *Taq* polymerase processing in the absence or presence of K^+^ and BRACO-19 (Figure 6C and D). The addition of the BRACO-19 ligand induced formation of *LTR-IV* only in the *WT* template and was found to stabilize *LTR-III* in both the *WT* and *G18T* templates. Interestingly, the *LTR-III* G-quadruplex was induced to a higher extent in the *G18T* template (Figure 6C and D).

Considering the dramatic effects of incorporating a single *G18T* base mutation on the HIV-1 promoter activity, we investigate the presence of such a mutation in about 1000 circulating viral strains available in the HIV sequence database (see ‘Materials and Methods’ section). We found a 99.5% degree of conservation for G18. There were five sequences observed to contain G18 mutations, four sequences contained a T mutation and one sequence an A at position G18 (Supplementary Tables S1 and 2). Collectively, these data support the crucial role of this position in virus replication and suggest that the *LTR-IV* G-quadruplex could act as a modulator of activity in the HIV-1 LTR promoter.

## DISCUSSION

We demonstrate here that the *LTR-IV* G-quadruplex adopts a well-defined parallel-stranded G-quadruplex containing a T-bulge (Figure 4). The single-nucleotide T19 bulge of *LTR-IV* participates in a stacking interaction with A17 of a neighboring single-nucleotide propeller loop. While the formation and prevalence of bulged G-quadruplexes have been previously described (57), to our knowledge, the loop-bulge interaction has not been reported so far. Although base stacking interactions between loops have previously been observed (58–60), the loop-bulge stacking and its orientation in the groove of the G-quadruplex may offer specificity in the design of a ligand specific to this structure.

It has been previously shown that the *LTR-IV* region is highly conserved (37). This is particularly true for the guanine bases within this sequence, suggesting the importance of conserving the ability for *LTR-IV* to form a G-quadruplex structure. In this work, mutation studies of the *LTR-IV* sequence demonstrate that the presence of a T-bulge is not crucial to G-quadruplex formation, but can play a role in modulating the G-quadruplex stability (57). However, at the proviral level the A17 and T19 nucleotides are within the Sp1 binding site, one of the most important transcription factors in the LTR promoter (61,62). Among 953 HIV-1 strains a mutation of A17 or T19 is observed in only 5 cases (1 case A17-to-G and 4 cases T19-to-G), supporting their key role in the virus life cycle. Mutation at G18, which disrupts *LTR-IV* G-quadruplex, is also shown to be very rare with a conservation rate of 99.5%. Collectively, the high conservation of these residues suggests that the bulge feature of the *LTR-IV* G-quadruplex is maintained across strains.

The LTR promoter of the proviral HIV-1 genome is crucial both for viral transcription and latency. In fact, it alone controls transcription of all nine viral genes, and repression of transcription at this level has been proposed to be involved in the switch to viral latency (63,64). It has been previously shown that the U3 region of the LTR can form G-quadruplexes and modulate viral transcription (44).

Our results suggest that G-quadruplex formation in *LTR-III* and *LTR-IV* may act as a regulatory mechanism for transcription activity, with *LTR-IV* formation acting as a negative regulator of *LTR-III*. This interpretation is based on the following. (i) We have shown here that when the capacity for *LTR-IV* to form a G-quadruplex is lacking (by *G18T* mutation), the LTR promoter activity is highly silenced in cells (Figure 6A). This effect is likely caused by the increase in *LTR-III* G-quadruplex formation, in addition to the disruption of one of the three binding sites of the transcription factor Sp1. In contrast, we have previously shown that mutations that prevent formation of *LTR-III* lead to enhanced LTR promoter activity (37). (ii) In the presence of BRACO-19, the *G18T* mutant, lacking *LTR-IV* quadruplex-formation capacity, leads to increased *LTR-III* G-quadruplex formation *in vitro* and decreased promoter activity in cells (Figure 6B and D).

We demonstrate that the *LTR-IV* G-quadruplex forms less readily than the *LTR-III* G-quadruplex, with a low population of the *LTR-IV* G-quadruplex forming in the full-length promoter (Figure 6C and D). Since the *LTR-IV* and *LTR-III* G-quadruplexes are mutually exclusive, our present data suggest that an increase of the *LTR-IV* G-quadruplex folding would reduce the amount of folded *LTR-III*. Based on our data, a disruption of the *LTR-IV* G-quadruplex formation reduced the LTR promoter activity, while a selective stabilisation of the *LTR-IV* G-quadruplex (e.g. via specific small molecules or proteins) would likely lead to increased transcriptional activity. A possible negative implication of over-active transcription could be premature cell death prior to completing virion production. Alternatively, over-active transcription could have ramifications on viral latency.

We propose the balance of *LTR-III* and *LTR-IV* G-quadruplex formation may act as a regulator of the viral promoter. The rational and selective targeting of the *LTR-IV* structure could lead to sharp effects on HIV-1 replication. Understanding the structures of LTR G-quadruplexes is a valuable first step in the rational design of a selective small molecule to target this region of the viral genome and modulate the transcriptional behavior of HIV.

## CONCLUSION

This work reports the NMR solution structure of the *LTR-IV* G-quadruplex formed in the LTR promoter of proviral HIV-1 genome. Biophysical data show that *LTR-IV* adopts a monomeric and parallel-stranded G-quadruplex in physiological solution. In the *LTR-IV* G-quadruplex, a single-nucleotide bulge interacts with a nearby single-nucleotide loop in a distinctive manner not reported previously. Conservation analysis shows these residues are highly conserved in viable HIV-1 strains. We suggest the loop-bulge interaction observed in *LTR-IV* is a promising feature that could be exploited in the design of molecules to specifically target this structure. The *LTR-IV* G-quadruplex structure presented here is valuable in understanding both the biology of the HIV-1 proviral LTR and its potential as a therapeutic target.

## ACCESSION NUMBER

PDB ID: 2N4Y.

## Supplementary Material

SUPPLEMENTARY DATA
