# Supplementary material for: Structure and possible function of a G-quadruplex in the long terminal repeat of the proviral HIV-1 genome
Source: Nucleic Acids Res. 2016 Jun 13;44(13):6442–51. doi: 10.1093/nar/gkw432 (PMC5291261; doi:10.1093/nar/gkw432)
Supplement: SUPPLEMENTARY DATA [file supp_44_13_6442__index.html]

Structure and possible function of a G-quadruplex in the long terminal repeat of the proviral HIV-1 genome — SUPPLEMENTARY DATA 

# Structure and possible function of a G-quadruplex in the long terminal repeat of the proviral HIV-1 genome

## SUPPLEMENTARY DATA

- SUPPLEMENTARY DATA
