# Supplementary material for: Structure and possible function of a G-quadruplex in the long terminal repeat of the proviral HIV-1 genome
Source: Nucleic Acids Res. 2016 Jun 13;44(13):6442–51. doi: 10.1093/nar/gkw432 (PMC5291261; doi:10.1093/nar/gkw432)

SUPPORTING INFORMATION

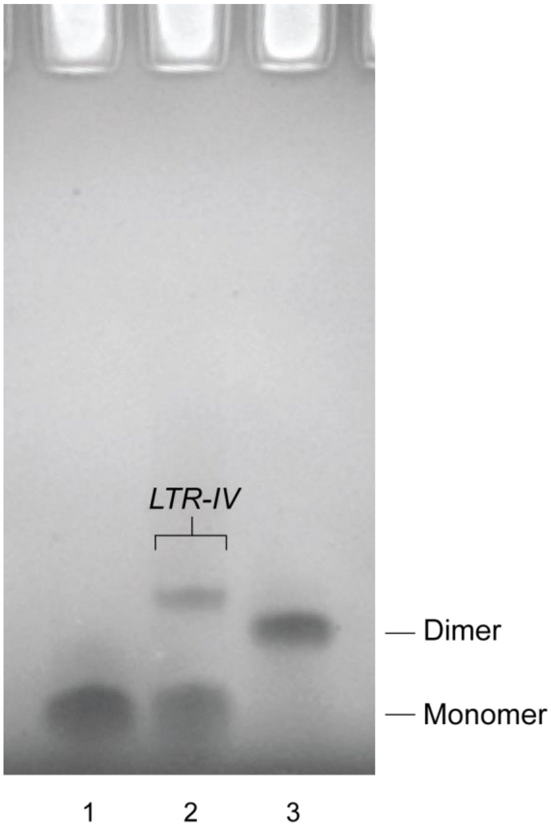

Figure S1. PAGE studies of *LTR-IV* (lane 2) in 10 mM KPi indicate a predominate monomeric G-quadruplex based on migration rates compared to a well-characterized monomeric parallel G-quadruplex formed from d[$T_2(G_3T)_4$] (lane 1) and a dimeric parallel G-quadruplex formed from d$(G_3T)_4$ (lane 3) (Do N.Q. & Phan A.T. (2012). *Chem. Eur. J.*, *18*, 14752.).

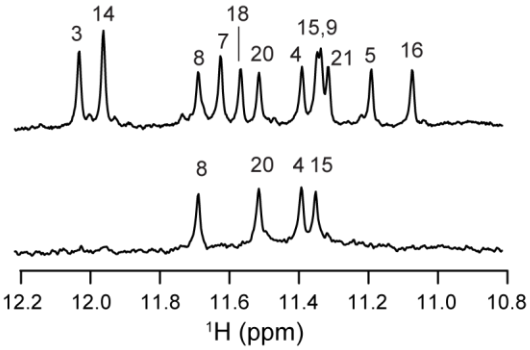

Figure S2. Solvent exchange experiments indicate the formation of a monomer G-quadruplex. Reference imino proton spectrum is shown on top. Imino proton spectrum after 60 minutes exposure to D_2O solvent is shown at the bottom.

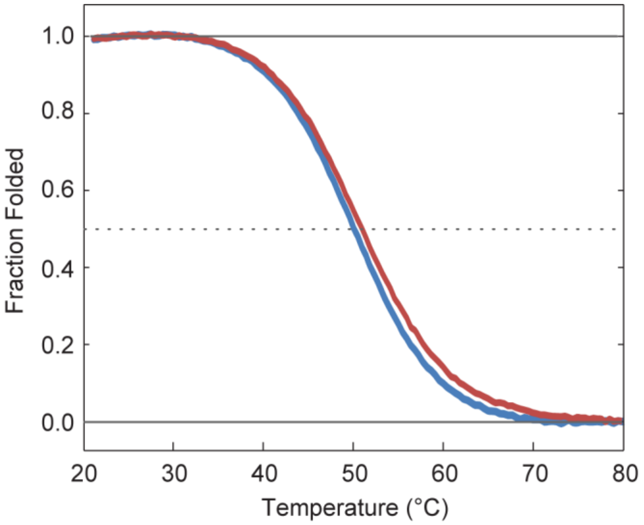

Figure S3. Thermal denaturing curve of the *LTR-IV* sequence in buffer containing 20 mM KPi (pH 7) and 70 mM KCl. The cooling curve (blue) and heating curve (red) are shown. The melting temperature (intersection with dotted black line) was calculated as the average of the two curves to be 50.5°C with a hysteresis of 0.9°C.

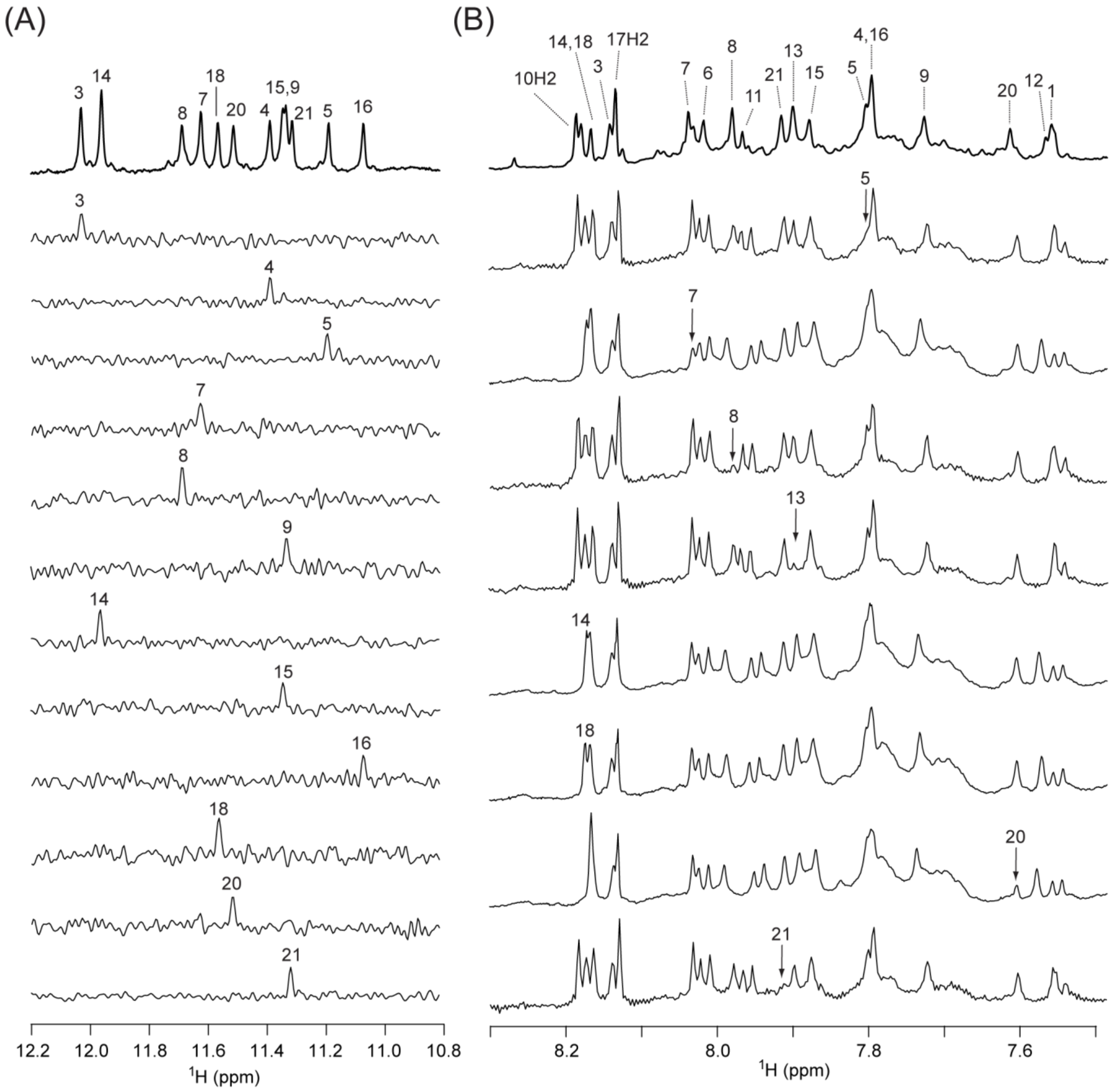

Figure S4. (A) Assignment of guanine imino (H1) protons using site-specific ^{15}N-enrichment (4%) and ^{15}N-filtered spectra carried out for all guanines within the G-tetrad core of the structure. The imino region of *LTR-IV* is shown as a reference. **(B)** Assignment of guanine aromatic (H8) protons using site-specific deuterium substitutions for specific guanines was performed by observing peaks that disappear upon substitution. The corresponding assigned spectral region of *LTR-IV* is shown as a reference.

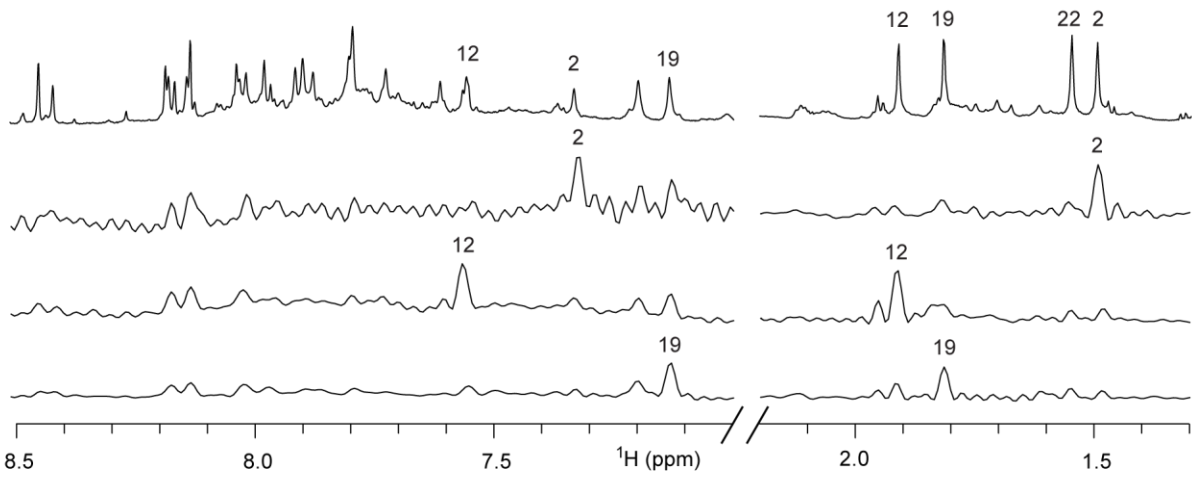

Figure S5. Assignment of thymine aromatic (left) and methyl (right) protons through ^{13}C-filtered experiments on sequenced containing site-specific ^{13}C-enriched thymine substitutions.

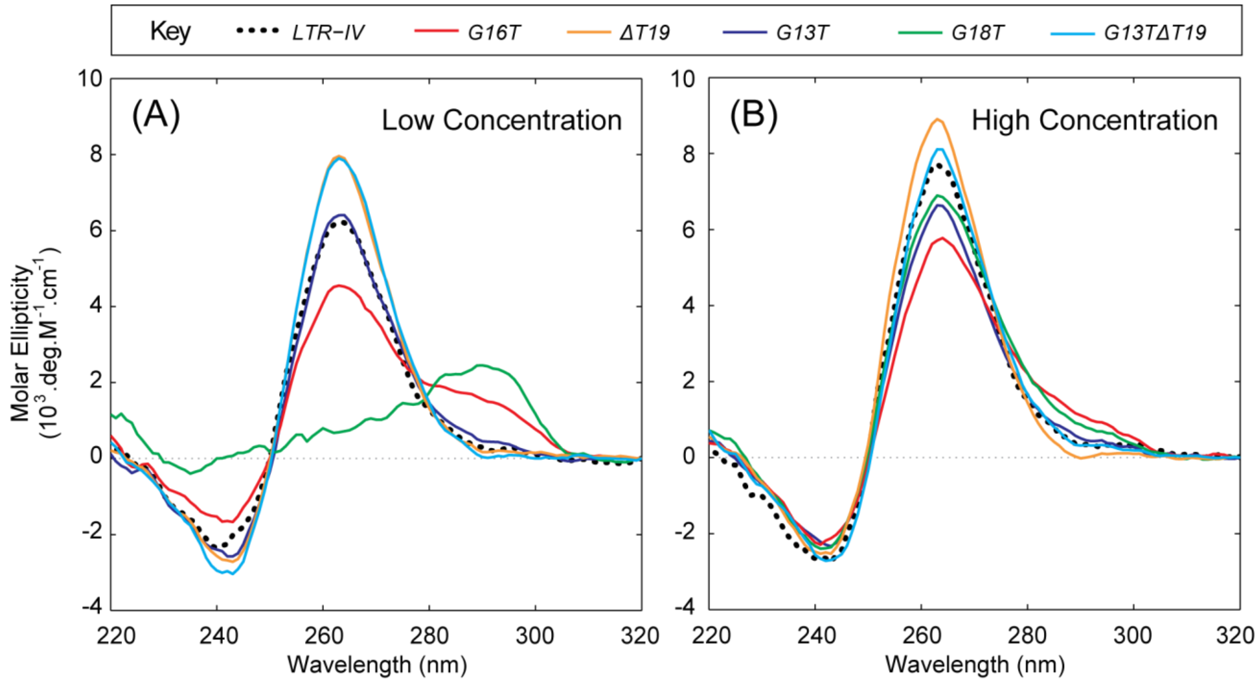

Figure S6. CD spectra of modified *LTR-IV* sequences in buffer containing 70 mM potassium chloride and 20 mM potassium phosphate (pH 7). **(A)** "Low Concentration" samples (oligo concentration of 5-10 µM) were annealed prior to CD measurements. The *G16T* and *G18T* sequences involve mutations to important guanine residues in the *LTR-IV* G-quadruplex. CD spectra of *G16T* and *G18T* show large deviation from the spectra of *LTR-IV*, suggesting the disruption of the native LTR-IV G-quadruplex. (B) "High Concentration" samples oligo concentration 250-500 µM) were annealed and then diluted to ~ 5 µM oligo concentration for CD measurements. All samples display behavior of a parallel G-quadruplex, suggesting a different form is adopted by some sequences when annealed at high concentration. This behavior is in line with high order band observed in gel electrophoresis of the *LTR-IV* sequence (Figure S1).

Table S1. HIV-1 strains mutated at position G18 among 953 naturally occurring HIV-1 strains.

	Accession	Subtype	Mutation at position G18
Ref.	NC_001802	B	-
1	AF063151	B	G → T
2	AF063152	B	G → T
3	AF127572	G5	G → T
4	AF196708	B	G → T
5	AF196709	B	G → A

Table S2. LTR Sequences of the 5 strains (Table S1) that are mutated at position 18. Shown is the G-rich region that can fold into G-quadruplex. The G18 position is indicated toward the 3' end with a bold square.

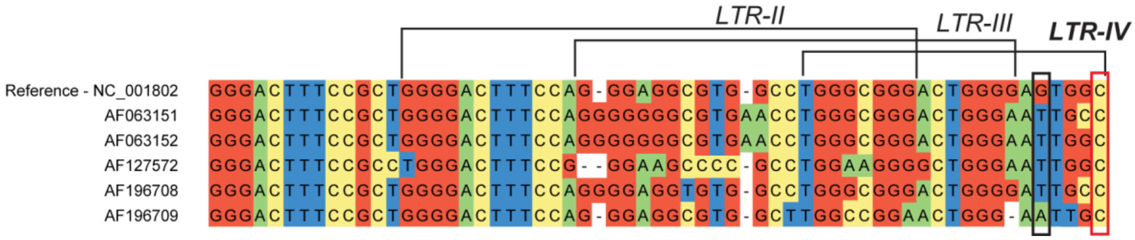

Supplement: SUPPLEMENTARY DATA [file supp_gkw432_SI_De_Nicola_et_al.pdf]
